# A similarity metric, rubric, and unified hierarchy for biomedical publication types and study designs

**DOI:** 10.1093/database/baag022

**Published:** 2026-06-05

**Authors:** Neil R Smalheiser, Joe D Menke, Arthur W Holt

**Affiliations:** Department of Psychiatry, University of Illinois Chicago, Chicago, IL, United States; School of Information Sciences, University of Illinois Urbana-Champaign, Champaign, IL, United States; Department of Psychiatry, University of Illinois Chicago, Chicago, IL, United States

## Abstract

Our goal is to unify the 72 biomedical publication types and study designs (collectively, PTs) into a single rubric and hierarchy. This is carried out in a data-driven manner by computing pairwise similarities of each PT against all others to form a similarity matrix. By performing hierarchical clustering we place each PT in a specific category and collect these into broader categories. Spearman correlations among PT pairs ranged from strongly negative to strongly positive (−0.732 to +0.997), with a mean of 0.176. Overall, we obtained 13 clusters of PTs and 5 more general categories: Observational Clinical Research, Qualitative and Genetic Methods, Clinical Evaluation and Validation, Interventional Trial Research, and Scholarly Synthesis and Discourse. These were then utilized to construct a unified hierarchy of PT terms. The rubric provides a flexible classification scheme for publication types and study designs that can accommodate new PTs as they are added over time. The similarity metric has the potential to improve the modelling, implementation, and evaluation of automated indexing systems. The PT rubric provides an overview that complements the existing NIH MeSH Hierarchy trees, and the unified hierarchy permits proper automated expansion for PT indexing and PubMed user queries involving PT terms.

## Introduction

Arguably the best curated indexing scheme for biomedical articles is the Medical Subject Heading (MeSH) terminology developed at the National Library of Medicine (NLM) [1]. Each article in MEDLINE is annotated with a set of 8–20 terms, according to the most important topics discussed. The terminology is arranged first in categories and then placed in a set of hierarchical trees, according to their logical relationships. One of the trees deals with Publication Types such as Review, Letter, or Editorial (https://www.nlm.nih.gov/mesh/pubtypes.html), and others include study design-related terms as well as topical terms. Until recently all indexing was performed by PhD-level curators who read the full text of each article and assign the most specific applicable term(s) on the hierarchy [2]. Currently, however, most indexing is carried out by automated machine learning systems [3]. The scheme is designed to facilitate high-recall retrieval of relevant articles when a user enters a free-text search query into the PubMed search interface. In the PubMed back-end, queries are processed to identify relevant MeSH terms and searches are automatically expanded to include synonyms and more specific terms that lie under them in the hierarchy [4].

In contrast to indexing of MeSH headings for the most important topics discussed in an article, some unique issues relate to automated indexing of articles in terms of publication types and study designs (e.g. case-control studies and cross-over studies) [5–8]. Our research team has been engaged in a long-term project along these lines [9–13]. We seek to tag each article explicitly with all indexing terms that apply to them. In addition, we recognize that some articles are atypical; thus, we do not simply generate binary yes/no predictions but also assign probabilistic scores for each term. Together, this strategy is designed to ensure that a user seeking to retrieve all articles of a given study design can identify them with high precision and recall, and further, can adjust the threshold of the probabilistic scores to either maximize precision or recall as desired [14, 15]. For simplicity, both publication types and study design-related terms will be collectively referred to as PTs.

During the course of our indexing project, we have identified several limitations regarding the NIH MeSH Hierarchy. First, the Publication Types form a separate tree apart from the study designs that are listed on multiple MeSH trees. Yet many Publication Types imply particular study designs; e.g. Randomized Controlled Trial is listed as a Publication Type but has definite design features (random allocation, comparing an experimental group against a control group, generally interventional, and often double-blind). Thus, it would be desirable to merge publication types and study design-related MeSH terms into a single rubric. Second, the relative position of two terms on the hierarchical trees does not necessarily provide a measure of how similar they are. A similarity metric would provide a fuller understanding of how PTs relate to each other.

### Objective

Our goal is to unify the multiple trees containing publication types and study designs into a single rubric and hierarchy. This is carried out in a data-driven manner by computing pairwise similarities of each PT against all others to form a similarity matrix. By performing hierarchical clustering, we place each PT in a specific category and collect these into broader categories.

## Materials and methods

In a previous publication-type indexing study [11], we processed the title and abstract of each article in the biomedical literature to create a vector representation of the article, then collected the vector representations of all articles belonging to a given PT and designated the centroid vector to represent the PT as a whole. In such a scheme, the similarity of two PTs can be measured in a straightforward manner as the distance between their centroids [16]. This metric was employed in a Support Vector Machine (SVM)-based predictive model of 50 PTs called Multi-Tagger [11]. However, more recently, we have utilized a transformer-based model that employed BERT-based encoders and represents articles not only in terms of text but some non-textual features as well (e.g. journal name, number of authors, and number of references) [12,13], and infers a probability value (0 < *P* < 1) that a given article belongs to each of 72 PTs. This model was employed here since it is more comprehensive and has been shown to have higher overall predictive performance for indexing PTs than the previous SVM-based model [12].

### Correlation analysis

The model probabilities produced by the transformer model were employed to obtain a pairwise similarity metric across all PTs. Full details of the model, its training data, and its architecture are given in Supplemental File 1. Briefly, the transformer model uses WeighCon supervised contrastive learning [17], and title, abstract, and various metadata-based features, such as journal name, served as input into the model. Training examples were taken from PubMed records published between 1987 and 2023 that had been manually assigned PTs by NLM, adjusting the number of examples per PT by under-sampling to deal with the fact that certain PTs have many more articles than others [12]. The model predicted both broad and narrow PTs simultaneously. For example, to retrieve articles having the ‘Clinical Study’ publication type, we made the following PubMed query: ((1987:2023[dp] AND (english[Language] OR english abstract[pt]) NOT indexingmethod_automated)) AND Clinical Study [pt] NOT editorial[pt] NOT letter[pt] NOT comment[pt] NOT practice guideline[pt] NOT review[pt]. Because of automatic PubMed query expansion, this strategy will retrieve not only articles whose XML metadata are directly indexed as ‘Clinical Study’, but all articles indexed by any publication type that lies below Clinical Study in the hierarchy (e.g. Clinical Trial and subtypes, Clinical Trial Protocol, and Observational Study). On the other hand, for publication types that are child nodes (i.e. have no other PTs below them), the PubMed queries retrieved only those articles having that PT indexed in the XML metadata.

In addition, using this same transformer model architecture, we added a new PT ‘Case Series’, designated from a curated corpus [18], combined with the data for the other PTs. This was motivated by the fact that NLM currently lacks formal definition and indexing for this type of publication. Both clinical case reports and case series are uncontrolled observational studies, but in general, a clinical case report is a detailed description of an incidental clinical encounter with one or a few patients. The case is sufficiently unique, rare, or interesting such that other medical professionals will learn something from it. In contrast, a case series is generally a planned systematic analysis (e.g. a retrospective chart review) of a larger set of patients, generally four or more (and sometimes hundreds). The two PTs are closely related (see below) but are not mutually exclusive, as a single article can satisfy both definitions, and historically NLM has indexed many case series with the case reports publication type. The transformer model predicted 2 537 196 PubMed articles as case reports and 99 915 as case series as of March 2026; only 7488 were assigned both PTs.

Overall, the transformer model was developed using a stratified random sample of 1 284 378 articles, which was stratified by PT to preserve label distribution. The dataset was split using 70/10/20 train/validation/test splits, again stratified to preserve label distribution in each split. Articles were tagged if the probabilistic prediction of the model was above a threshold, which was determined empirically to optimize for F1 within the validation set. The test set of articles from the modelling work, containing 256 897 articles, formed the basis for the present analysis and was further filtered to include only articles with complete abstracts, leaving 210 641 articles for analysis. (Articles lacking abstracts were excluded because they have limited textual features and on average show lesser predictive accuracy [12].)

**Figure 1:**
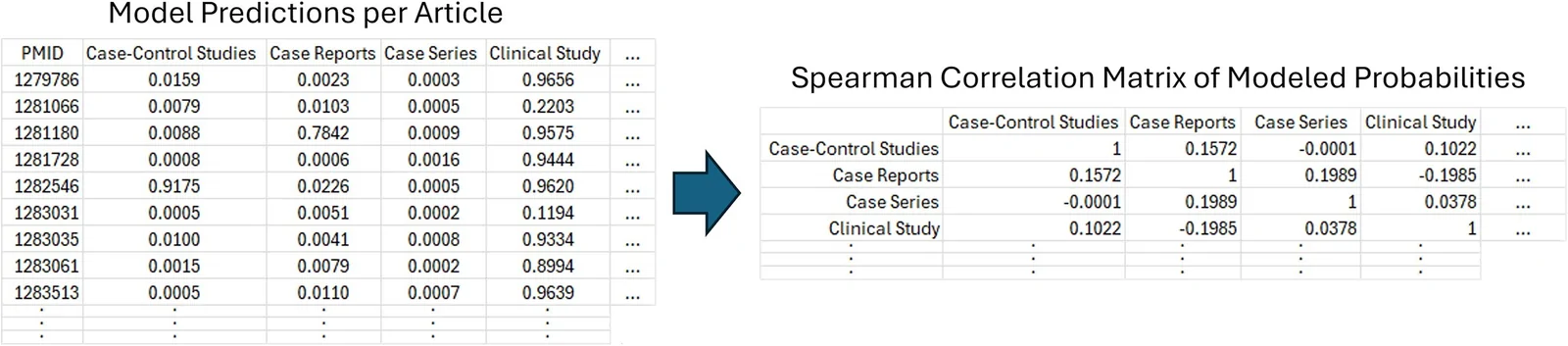
Correlation matrix derivation. Modelled predicted probabilities were used to compute Spearman correlations for each pairwise combination of publication types.

From the model-predicted probabilities for each of the 72 publication types across the articles of the test set, a non-linear (Spearman) correlation ρ was computed for all 2556 pairwise combinations of the 72 publication types (illustrated in Fig. 1) to form a 72 × 72 correlation matrix (Supplemental File 2).

## Results

### Distribution of PT similarity measures

**Figure 2:**
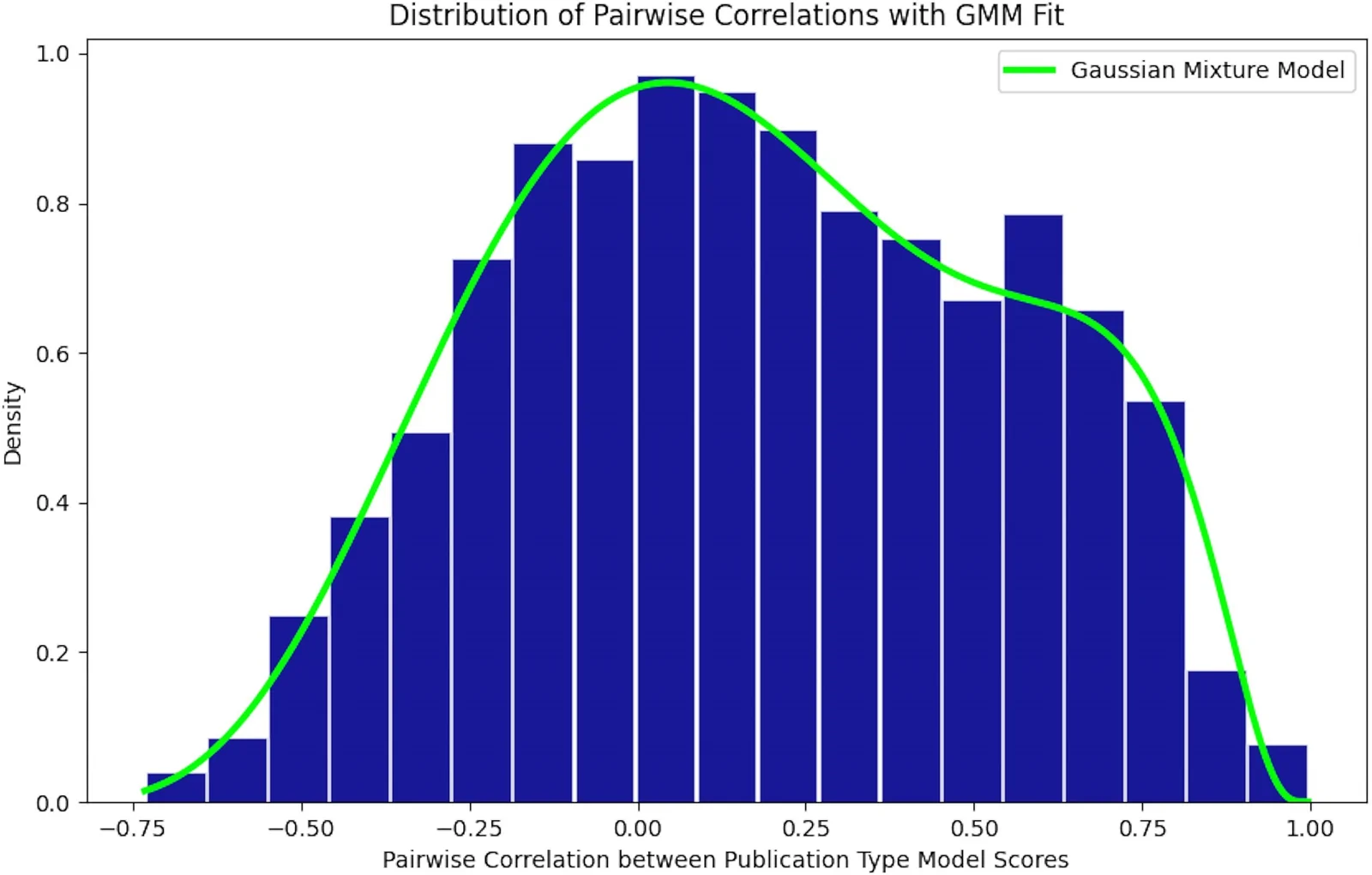
Distribution of pairwise correlations for PTs. Spearman correlation was computed among model-predicted probabilities for assignment of 72 publication types forming a 72 × 72 correlation matrix. The distribution of the correlation coefficients is bimodal with one mode centred around zero, the other around 0.57.

Model score correlations among PT pairs ranged from strongly negative to strongly positive (−0.732 to +0.997), with a mean of 0.176. The distribution appears to be bimodal with one mode centred near zero correlation, and a shoulder centred around 0.59 (Fig. 2). The highest positive correlations relate to clinical trial subtypes and related clinical study methodologies. The negative correlations consist of publication type pairs that are functionally unrelated, such as Clinical Study vs. Biography. It should be pointed out that a pair of PTs may show partial correlations either due to semantic similarity (e.g. shared article vocabularies) or due to inconsistencies in NLM indexing, in which a given type of article may sometimes receive one PT and sometimes another similar one (e.g. Personal Narrative vs. Autobiography). The observed clustering is robust, in the sense that if we perturb the set of 72 correlations by random choice of −x%, 0, +x% for percentages ranging from 1% to 2.5% of the correlation value (in increments of 0.1%), the identification of the nearest-neighbour PT was totally stable when perturbations were repeated 2000 times for each value of x; for each x we also shuffled the order of PTs to be clustered 100 times, giving a total of 200 000 runs. In other words, the choice of nearest-neighbour PT for any PT pair was not influenced by small changes in the exact value of the Spearman correlation, nor in the order in which PTs were listed and handled.

### Hierarchical clustering of publication types and study designs

**Figure 3:**
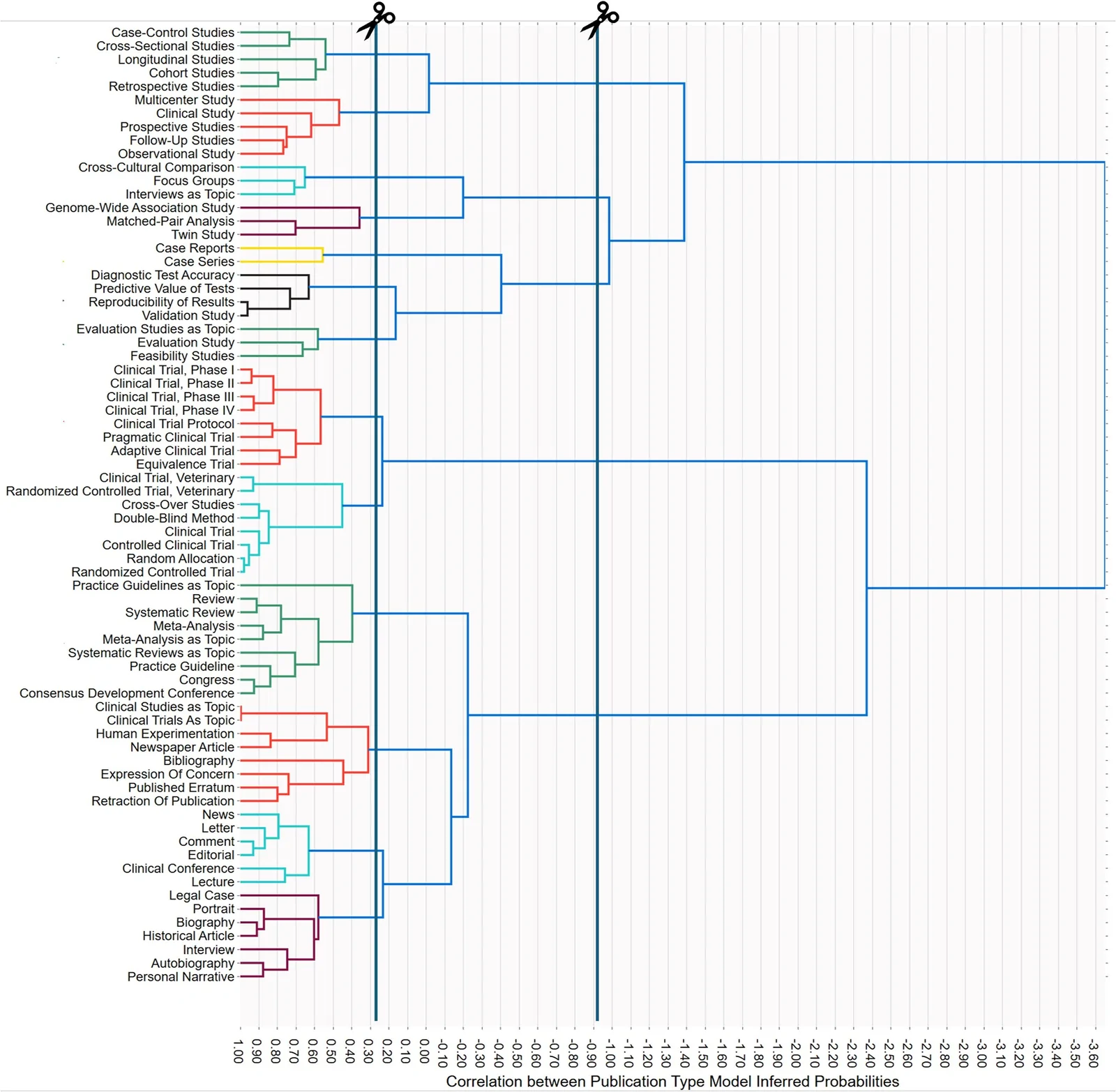
Hierarchical clustering of publication types by model-inferred probabilities. Publication type clusters derived from pairwise model score correlations and grouped with hierarchical clustering. Cut points were chosen to delineate 13 low-level categories and 5 broader categories.

Several different clustering techniques were evaluated, including graph-based community detection methods and statistical clustering methods. The graph-based clustering methods produced reasonable communities, but were found to be sensitive to noise and joined disparate publication types such as case reports with editorials and commentary. Agglomerative hierarchical clustering provided a more reasonable and functional basis for grouping PTs using the pairwise correlation matrix inverted to represent distances and joined with Ward linkage [19] (Fig. 3).

Starting with the most similar pairs of PTs, several different categories naturally emerged in a data-driven manner (Fig. 3). For example, the category of Observational Epidemiologic Study Designs comprised case-control, cross-sectional, longitudinal, cohort, and retrospective studies. Interestingly, other types of observational studies fell into a distinct category, General Clinical and Observational Studies, comprising terms residing on several different MeSH Hierarchy trees, namely, multicentre study, clinical study, prospective studies, follow-up studies, and observational study. The reason for this split reflects the behaviour of NLM indexers, who attach only the most specific indexing term that applies to a given article to its XML metadata. Thus, even though the definition of Clinical Study comprises both interventional and observational designs (https://www.ncbi.nlm.nih.gov/mesh/2009830), if the article is interventional, NLM indexers would affix only the more specific term Clinical Trial instead. This means that Clinical Trial (logically a subset of Clinical Study) is placed with interventional designs, whereas NLM indexers would affix the term ‘Clinical Study’ only if it is NOT a clinical trial. The relationships shown in Fig. 3 are thus complementary to the hierarchical logical relationships and trees displayed in the NIH Hierarchies.

Overall, we obtained 13 low-level clusters of PTs and 5 more general categories: Observational Clinical Research, Qualitative and Genetic Methods, Clinical Evaluation and Validation, Interventional Trial Research, and Scholarly Synthesis and Discourse. One PT, Scientific Integrity Review, was excluded from the clustering process because very few articles in our dataset were tagged with that type; due to its nature (consisting of official scientific misconduct findings), it was manually placed in Scholarly Publishing and Research Integrity. Table 1 contains a full list of publication types and study designs examined along with low-level and broader category assignments. Another view is presented in Supplemental File 2.

**Table 1:** Publication types and observed cluster assignments.

Publication type	Low-level category	Broader category
Case-Control Studies Cross-Sectional Studies Longitudinal Studies Cohort Studies Retrospective Studies	Observational Epidemiologic Study Designs	Observational Clinical Research
Multicentre Study Clinical Study Prospective Studies Follow-Up Studies Observational Study	General Clinical & Observational Studies	Observational Clinical Research
Cross-Cultural Comparison Focus Groups Interviews as Topic	Qualitative & Sociocultural Research Methods	Qualitative & Genetic Methods
Genome-Wide Association Study Matched-Pair Analysis Twin Study	Genetic & Matched Population Analyses	Qualitative & Genetic Methods
Case Reports Case Series	Clinical Case-Based Evidence	Clinical Evaluation & Validation
Diagnostic Test Accuracy Predictive Value of Tests Reproducibility of Results Validation Study	Diagnostic & Methodological Validation Studies	Clinical Evaluation & Validation
Evaluation Studies as Topic Evaluation Study Feasibility Studies	Program & Process Evaluation Studies	Clinical Evaluation & Validation
Clinical Trial, Phase I Clinical Trial, Phase II Clinical Trial, Phase III Clinical Trial, Phase IV Clinical Trial Protocol Pragmatic Clinical Trial Adaptive Clinical Trial Equivalence Trial	Interventional Clinical Trial Phases & Designs	Interventional Trial Research
Clinical Trial, Veterinary Randomized Controlled Trial, Veterinary Cross-Over Studies Double-Blind Method Clinical Trial Controlled Clinical Trial Random Allocation Randomized Controlled Trial	Controlled & Randomized Trial Methodology	Interventional Trial Research
Practice Guidelines as Topic Review Systematic Review Meta-Analysis Meta-Analysis as Topic Systematic Reviews as Topic Practice Guideline Congress Consensus Development Conference	Evidence Synthesis & Clinical Guidance	Scholarly Discourse and Evidence Synthesis
News Letter Comment Editorial Clinical Conference Lecture	Scientific Commentary & Professional Discourse	Scholarly Discourse and Evidence Synthesis
Legal Case Portrait Biography Historical Article Interview Autobiography Personal Narrative	Biographical, Historical & Narrative Works	Scholarly Discourse and Evidence Synthesis

### A unified hierarchy of publication types and study designs

The rubric presented so far merges PT terms that reside across multiple trees in the NLM MeSH hierarchy. However, it still remains to organize the terms into a single hierarchy that reflects their logical relationships, which is necessary in order to perform proper automated expansion for PT indexing and retrieval of user queries. We constructed the hierarchy manually, being informed by three sources of evidence: (a) maintaining the existing MeSH Hierarchy placements whenever possible, (b) considering the nearest-neighbour PTs and rubric categories (Fig. 3 and Table 1), and (c) examining the automated query expansion of terms as performed by the PubMed search engine. The proposed unified hierarchy is shown in Table 2.

**Table 2:** Unified hierarchy of publication types and study designs.^a^

**Clinical Evaluation & Validation** *Diagnostic & Methodological Validation Studies* Diagnostic Test Accuracy Predictive Value of Tests Reproducibility of Results Validation Study *Program & Process Evaluation Studies* Evaluation Studies as Topic Evaluation Study Feasibility Studies **Multicentre Study** **Clinical Study** *Retrospective Studies* *Prospective Studies* *Clinical Trial* Controlled Clinical Trial Randomized Controlled Trial Equivalence Trial Clinical Trial, Phase I Clinical Trial, Phase II Clinical Trial, Phase III Clinical Trial, Phase IV Clinical Trial Protocol Pragmatic Clinical Trial Adaptive Clinical Trial Cross-Over Studies Double-Blind Method Random Allocation Clinical Trial, Veterinary Randomized Controlled Trial, Veterinary *Observational Study* Case-Control Studies Cross-Sectional Studies Longitudinal Studies Cohort Studies Follow-Up Studies Case Reports Case Series **Qualitative & Genetic Methods** *Genetic & Matched Population Analyses* Genome-Wide Association Study Matched-Pair Analysis Twin Study *Qualitative & Sociocultural Research Methods* Cross-Cultural Comparison Focus Groups Interviews as Topic
**Scholarly Discourse and Evidence Synthesis** *Biographical, Historical & Narrative Works* Historical Article Biography Autobiography Interview Personal Narrative Legal Case Portrait *Evidence Synthesis & Clinical Guidance* Practice Guidelines as Topic Review Systematic Review Meta-Analysis Meta-Analysis as Topic Systematic Reviews as Topic Practice Guideline Congress Consensus Development Conference *Scholarly Publishing & Research Integrity* Clinical Studies as Topic Clinical Trials As Topic Human Experimentation Newspaper Article Bibliography Expression of Concern Published Erratum Retraction of Publication Scientific Integrity Review *Scientific Commentary & Professional Discourse* News Letter Comment Editorial Clinical Conference Lecture

a Level 1 terms are indicated in bold; level 2 terms in italics. Terms that correspond to rubric categories are in black, whereas MeSH Hierarchy terms are in purple. Included here are two non-standard terms not found on the MeSH Hierarchy that have been modelled by our group (diagnostic test accuracy and case series) [11, 18].

The placement of several PT terms in Table 2 deserves notice. (1) ‘Clinical Trial Protocol’ is indexed under ‘Clinical Study’ in the PT Hierarchy. We kept this arrangement in our unified hierarchy, but note that protocol articles should arguably also be included under the ‘Clinical Trials as Topic’ MeSH term on historical as well as logical grounds, since protocols are articles about clinical trials and thus satisfy the broader MeSH term. The Clinical Trial Protocol term was introduced as a distinct PT only recently, in 2019, so earlier protocol articles were presumably indexed under ‘as Topic’. (3) All observational study designs are now placed under ‘Observational Study’; this implies that all articles having observational designs (e.g. cohort studies) should automatically be assigned Observational Study as well. Note that this is not currently carried out by PubMed via automated query expansion, as Observational Study resides on a different tree than the individual study designs. (4) ‘Retrospective Studies’ and ‘Prospective Studies’ are no longer placed under either ‘Cohort Studies’ or ‘Case-Control Studies’, but are placed directly under Clinical Study. This corrects a major anomaly in the existing MeSH Hierarchy, since many articles with retrospective or prospective designs are actually neither cohort studies nor case-control studies. In fact, although the existing MeSH Hierarchy considers both Retrospective and Prospective as observational study designs, a minority (∼20%) of articles indexed by NLM as Retrospective or Prospective are dually indexed as Clinical Trial. Because these study designs can be either observational or interventional, we placed Retrospective and Prospective designs directly under Clinical Study.

## Discussion

The formulation of a similarity metric for PTs led to the construction of a rubric that provides a simple, concrete overview of the many biomedical publication types and study designs, in a single diagram. This provides a complementary view from the NIH hierarchies which identify different terms in terms of their logical relationships and proximity on several independent trees. Cid and Mork previously constructed a PT correlation matrix based on the frequency of co-occurrence of multiple PTs appearing on individual articles [8], but their scheme did not include study design-related MeSH terms and only considers articles that received multiple PT terms. In contrast, our similarity metric was computed across all articles and reflects the indexing decisions of NLM indexers, who generally attach only the most specific indexing term that applies to a given article. Hence, our scheme provides a more comprehensive, finer-grained overview that can be applied to new PTs not indexed by NLM (e.g. Case Series) as well as to pairs of PTs that co-occur rarely or not at all on individual articles.

As the similarity metric is data-driven, it can be recalculated to adapt to future changes in publications, and can easily accommodate new indexing terms as they arise over time. For example, since NLM currently has no formal indexing of case series articles, we recently created a new publication type, Case Series [18]. After adding this PT to the existing transformer model, we obtained correlation values which placed Case Series next to Case Reports (Fig. 2).

Apart from providing a classification scheme for publication types and study designs, we believe that the similarity metric should have utility for better automated PT indexing. With regard to the current transformer model, we use a one-against-all model in which each article is assessed for a given PT vs. all others. However, the training process should be sharpened if one used training examples of one PT as negative examples against another PT that is very similar (but not a subset of the other). For example, Case Reports and Case Series are nearest-neighbour PTs in the rubric. Case reports tend to be incidental observations of one or a few patients, whereas case series tend to be planned observations of four or more patients, but the two types of article are not sharply delineated [18]. By comparing and contrasting these two PTs during model training, the probabilistic scores should better discriminate whether a given article has typical case report-like vs. typical case series-like features.

The similarity metric may also assist in evaluating the errors made by an automated indexing system. Since the probabilistic predictive scores for nearest-neighbour PTs have high Spearman correlation values, one would expect that a mis-assigned article would most likely be tagged with the nearest-neighbour PT instead. In many cases, the errors might be of little import (e.g. mis-assigning a Focus Group article as Interviews as Topic). Other nearest-neighbour errors have high importance for high recall retrieval, e.g. mis-assigning Cohort Studies as Case-Control Studies. Again, identifying these cases would prioritize them for contrastive training in which one PT is used as negative examples for the other PT.

Another possible use for the rubric is as a framework for alternative Large Language Model (LLM)-based assignment of publication types and study designs [6]. For example, the organization of PTs into small and larger categories allows one to envision a Twenty Questions procedure in which the LLM would first attempt to place an article into the most appropriate broad category (e.g. observational vs. interventional research) and then to ascertain the best low-level category and the best choice among the PTs in that category.

This study has several limitations. The processing of articles was limited to articles containing abstracts, which provide more complete data for model predictive scores; this will under-sample a few PTs that have a high proportion of articles lacking abstracts (e.g. Letter or Portrait). However, these PTs tend to be the least important in terms of clinical relevance and should have little effect on the rubric as a whole. Another limitation is that agglomerative hierarchical clustering’s optimal solution may be expected to differ slightly, e.g. across different examined time periods, as the biomedical literature evolves and changes. Regardless, the placement of PTs into categories is likely to be robust and stable over time. We confirmed that the clustering was robust to small changes in the exact value of the Spearman correlations or in the order of handling the list of PTs.

Finally, the unified rubric informed the construction of a single PT hierarchy, that merges PT terms across multiple trees of the MeSH Hierarchy and corrects several of its deficiencies and anomalies (Table 2). The new hierarchy permits better automated expansion for PT indexing; e.g. all observational study designs are now automatically assigned Observational Study as well.

We propose that this hierarchy is also preferred for PubMed user queries, for automated expansion of PT terms. We are currently applying our transformer model to process all articles indexed by PubMed and generate predictive scores for 72 publication types and study designs. The scores will be disseminated publicly on our project website (https://arrowsmith.psych.uic.edu). To convert predictive scores to binary yes/no predictions, we first apply score thresholds corresponding to the best F1 on a validation set (as in [9, 11]), and then enforce post-prediction normalization using the unified hierarchy. For example, all articles whose predictive scores for Cohort Studies are above threshold will be tagged as Cohort Studies, but will also automatically be tagged as Clinical Study (regardless of their Clinical Study predictive scores). This will ensure greater recall and consistency of indexing for terms that are in the upper levels of the hierarchy.

## Conclusion

We present here pairwise similarity measures that allowed us to construct a rubric that arranges publication types and study designs in a unified diagram and a unified hierarchy. Such a rubric complements the existing NIH hierarchies and has the potential to improve the modelling, implementation, and evaluation of automated indexing systems.

## Supplementary Material

baag022_Supplemental_Files

## Data Availability

The non-linear (Spearman) correlation ρ was computed for all pairwise combinations of the 72 publication types (illustrated in Fig. 1) to form a 72 × 72 correlation matrix. This is shown in Supplemental File 2. The unified hierarchy is presented in computable JSON format in Supplemental File 3.
