# Supplementary material for: A similarity metric, rubric, and unified hierarchy for biomedical publication types and study designs
Source: Database (Oxford). 2026 Jun 5;2026:baag022. doi: 10.1093/database/baag022 (PMC13237591; doi:10.1093/database/baag022)
Supplement: baag022_Supplemental_Files [file baag022_supplemental_files.zip › Supplemental File 3.docx]

**Supplemental File 3.** The unified hierarchy presented in computable JSON format.

#########start

{

  "Clinical Evaluation & Validation": { "node_type": "category",

    "children": { "Diagnostic & Methodological Validation Studies": { "node_type": "category",

        "children": { "Diagnostic Test Accuracy": { "node_type": "pt" },

          "Predictive Value of Tests": { "node_type": "pt" },

          "Reproducibility of Results": { "node_type": "pt" },

          "Validation Study": { "node_type": "pt" } } },

      "Program & Process Evaluation Studies": { "node_type": "category",

        "children": { "Evaluation Studies as Topic": { "node_type": "pt" },

          "Evaluation Study": { "node_type": "pt" },

          "Feasibility Studies": { "node_type": "pt" } } } } },

  "Multicenter Study": { "node_type": "pt" },

  "Clinical Study": { "node_type": "pt",

    "children": { "Retrospective Studies": { "node_type": "pt" },

      "Prospective Studies": { "node_type": "pt" },

      "Clinical Trial": { "node_type": "pt",

        "children": {

          "Controlled Clinical Trial": { "node_type": "pt",

            "children": {"Randomized Controlled Trial": { "node_type": "pt",

              "children": {"Equivalence Trial": { "node_type": "pt" }

                }

              }

            }

           }

          ,

          "Clinical Trial, Phase I": { "node_type": "pt" },

          "Clinical Trial, Phase II": { "node_type": "pt" },

          "Clinical Trial, Phase III": { "node_type": "pt" },

          "Clinical Trial, Phase IV": { "node_type": "pt" },

          "Clinical Trial Protocol": { "node_type": "pt" },

          "Pragmatic Clinical Trial": { "node_type": "pt" },

          "Adaptive Clinical Trial": { "node_type": "pt" },

          "Cross-Over Studies": { "node_type": "pt" },

          "Double-Blind Method": { "node_type": "pt" },

          "Random Allocation": { "node_type": "pt" },

          "Clinical Trial, Veterinary": { "node_type": "pt",

            "children": {"Randomized Controlled Trial, Veterinary": { "node_type": "pt" } }

         }

        }

      },

      "Observational Study": { "node_type": "pt",

        "children": { "Case-Control Studies": { "node_type": "pt" },

          "Cross-Sectional Studies": { "node_type": "pt" },

          "Longitudinal Studies": { "node_type": "pt" },

          "Cohort Studies": { "node_type": "pt" },

          "Follow-Up Studies": { "node_type": "pt" },

          "Case Reports": { "node_type": "pt" },

          "Case Series": { "node_type": "pt" } } } }

        },

  "Qualitative & Genetic Methods": { "node_type": "category",

    "children": { "Genetic & Matched Population Analyses": { "node_type": "category",

        "children": { "Genome-Wide Association Study": { "node_type": "pt" },

          "Matched-Pair Analysis": { "node_type": "pt" },

          "Twin Study": { "node_type": "pt" } } },

      "Qualitative & Sociocultural Research Methods": { "node_type": "category",

        "children": { "Cross-Cultural Comparison": { "node_type": "pt" },

          "Focus Groups": { "node_type": "pt" },

          "Interviews as Topic": { "node_type": "pt" } } } } },

  "Scholarly Discourse and Evidence Synthesis": { "node_type": "category",

    "children": { "Biographical, Historical & Narrative Works": { "node_type": "category",

        "children": { "Historical Article": { "node_type": "pt" ,

          "children": {

            "Biography": { "node_type": "pt",

              "children" : {"Autobiography": { "node_type": "pt" }}

              }

          }

        },

          "Interview": { "node_type": "pt" },

          "Personal Narrative": { "node_type": "pt" },

          "Legal Case": { "node_type": "pt" },

          "Portrait": { "node_type": "pt" } } },

      "Evidence Synthesis & Clinical Guidance": { "node_type": "category",

        "children": { "Practice Guidelines as Topic": { "node_type": "pt" },

          "Review": { "node_type": "pt" },

          "Systematic Review": { "node_type": "pt" },

          "Meta-Analysis": { "node_type": "pt" },

          "Meta-Analysis as Topic": { "node_type": "pt" },

          "Systematic Reviews as Topic": { "node_type": "pt" },

          "Practice Guideline": { "node_type": "pt" },

          "Congress": { "node_type": "pt" },

          "Consensus Development Conference": { "node_type": "pt" } } },

      "Scholarly Publishing & Research Integrity": { "node_type": "category",

        "children": { "Clinical Studies as Topic": { "node_type": "pt" },

          "Clinical Trials As Topic": { "node_type": "pt" },

          "Human Experimentation": { "node_type": "pt" },

          "Newspaper Article": { "node_type": "pt" },

          "Bibliography": { "node_type": "pt" },

          "Expression Of Concern": { "node_type": "pt" },

          "Published Erratum": { "node_type": "pt" },

          "Retraction Of Publication": { "node_type": "pt" },

          "Scientific Integrity Review": { "node_type": "pt" } } },

      "Scientific Commentary & Professional Discourse": { "node_type": "category",

        "children": { "News": { "node_type": "pt" },

          "Letter": { "node_type": "pt" },

          "Comment": { "node_type": "pt" },

          "Editorial": { "node_type": "pt" },

          "Clinical Conference": { "node_type": "pt" },

          "Lecture": { "node_type": "pt" } } } } }

}

########## end
